# I, robot: depression plays different roles in human–human and human–robot interactions

**DOI:** 10.1038/s41398-021-01567-5

**Published:** 2021-08-21

**Authors:** Dandan Zhang, Junshi Shen, Sijin Li, Kexiang Gao, Ruolei Gu

**Affiliations:** 1 School of Psychology, Shenzhen University, 518060 Shenzhen, China; 2 Magnetic Resonance Imaging Center, Shenzhen University, 518060 Shenzhen, China; 3 CAS Key Laboratory of Behavioral Science, Institute of Psychology, 100101 Beijing, China; 4 Department of Psychology, University of Chinese Academy of Sciences, 100049 Beijing, China

**Keywords:** Human behaviour, Prognostic markers

## Abstract

Socially engaging robots have been increasingly applied to alleviate depressive symptoms and to improve the quality of social life among different populations. Seeing that depression negatively influences social reward processing in everyday interaction, we investigate this influence during simulated interactions with humans or robots. In this study, 35 participants with mild depression and 35 controls (all from nonclinical populations) finished the social incentive delay task with event-related potential recording, in which they received performance feedback from other persons or from a robot. Compared to the controls, the mild depressive symptom (MDS) group represented abnormalities of social reward processing in the human feedback condition: first, the MDS group showed a lower hit rate and a smaller contingent-negative variation (correlated with each other) during reward anticipation; second, depression level modulated both the early phase (indexed by the feedback-related negativity (FRN)) and the late phase (indexed by the P3) of reward consumption. In contrast, the effect of depression was evident only on FRN amplitude in the robot feedback condition. We suggest that compared to human–human interaction, the rewarding properties of human–robot interaction are less likely to be affected by depression. These findings have implications for the utilization of robot-assisted intervention in clinical practice.

## Introduction

Social interaction is essential to people’s livelihood, career development, mental health, and psychological well-being. In human beings, one of the major motivators for social interaction is to pursue social rewards [1, 2]. Here, “social reward” refers to social information that has rewarding properties, including social approval, social belonging, and social support [3]. Depression is closely associated with the abnormal processing of social rewards at both behavioral and neural levels [4]. Severe expression of depression symptoms and syndromes has been linked to blunted response to social rewards (i.e., social anhedonia) [5–7]. Depression severity negatively correlates with the activation level of the human reward system, including dopaminergic neural circuits [8–11]. Consequently, these problems in social reward processing inhibit depressed individuals’ motivation to engage in social interaction [12], which could help understand the impairments of social functioning in depression [13].

Nowadays, human-like robots have been prevailing in daily life, many of which are specifically designed to establish social relations with humans (i.e., artificially intelligent social machines) [14]. These socially engaging artificial agents have been increasingly employed for psychosocial intervention on depression [15]. Using social robots for clinical practice has important implications, seeing that some populations (e.g., older adults and disabled persons) need to maintain a certain degree of social interaction and engagement, but medical resources for providing social support are often limited [16]. Some recent studies have shown that interacting with social robots has promising effects on geriatric depression and counteracting feelings of loneliness [17–21]. Considering these new developments, a question that emerges is whether social reward dysfunctions in depression could influence not only human–human interaction but also human–robot interaction, which is the main interest of this study. Investigating this topic would help determine: (1) the value of a robot-assisted intervention as a means of nonpharmacological treatment, and (2) the nature of social interaction from a theoretical point of view [14].

Interacting with an artificial entity is like interacting with fellow humans in many aspects. First, people are willing to follow their usual habits when communicating with human-like agents, using social skills, such as gestures and facial signals [22–24]. Second, people are prone to apply the same social rules and expectations to automations as they do to humans, indicating that social psychological knowledge derived from human–human interaction also guides human–machine interaction [25–28]. For example, people can “recognize” personality cues from computer-synthesized speech [29]. Likewise, De Kleijn et al. found that participants in the classic Ultimatum Game allocate an equal amount of resources to human and robot opponents [30–33]. Human faces and humanoid faces are equally likely to trigger an automatic orientation of attention [34], and they both activate face-responsive brain regions including the fusiform gyrus and superior temporal sulcus as well as the mirror neuron system [35]. These phenomena could be regarded as manifestations of anthropomorphism, that is, the tendency to attribute human characteristics to non-human entities—especially those having human-like appearances [36, 37]. Therefore, it is understandable that communications with artificial agents could be perceived to have social rewarding properties [38, 39]. For instance, “friendly” computer feedback is evaluated as more favorable than “unfriendly” feedback by individuals [25]. Also, happy compared to neutral expressions of both human and robot faces shortened individual reaction time (RT) and enhanced the P1 component of event-related potentials (ERPs) [40–42]. People may even develop a long-term “parasocial” relationship with their companion robots (e.g., at health service facilities) [43–46].

To our knowledge, no study to date has directly addressed whether depression would impair the social rewarding nature of human–robot interaction. However, this possibility is indicated by the research focusing on other types of mental disorders: for instance, Raffard et al. discovered that negative symptoms of schizophrenia negatively affect the accuracy of facial expression discrimination in both human and robotic conditions [38, 47]. In this study, we used the classic social incentive delay (SID) task designed by Spreckelmeyer et al. to investigate social reward processing [48, 49]. In each trial of the SID task, participants observe an incentive cue indicating the amount of potential reward, then respond to a target stimulus as quickly as possible, and finally receive social feedback (e.g., a smiling face or a “thumbs-up” gesture) as rewards according to their performance. Here, a major advantage of the SID task is that it enables to discriminate between the anticipation stage (corresponding to cue presentation) and the consumption stage (corresponding to feedback presentation) of social reward processing [50, 51]. Using this paradigm, recent studies have revealed that depressive symptoms are associated with deficits in both stages [52–55].

In order to observe not only behavioral but also neural manifestations of reward processing abnormalities, this study relies on the ERPs that are suitable to reflect the dynamics of brain activity over the time course of reward processing [56–59]. Combining the SID task with the ERP technique has been proven to be successful [60–62]. According to our research interest, we focus on three ERP components, including the contingent-negative variation (CNV) associated with cue presentation, and the feedback-related negativity (FRN) and P3 associated with feedback presentation. The CNV is a frontocentral distributed negativity that is elicited by a preceding signal, indicating preparation processes for an upcoming event [63, 64]. In the SID context, the CNV elicited by cue presentation indicates the anticipation of the reward-related target [61]. The FRN is another frontocentral distributed negativity in response to feedback stimulus, being larger for unfavorable than favorable feedback [65–67]. It is possible that the FRN indicates a quick, coarse, and bottom-up detection of feedback value [68–70]. Following the FRN, the P3 is also an important feedback-locked component that is positive-going and centro-parietal distributed [71, 72]. This component is suggested to reflect a more deliberate, comprehensive, top-down evaluation of feedback information [73–75]. According to previous studies, the amplitudes of these ERP components are all sensitive to individual depression level [10, 11, 76, 77]. Most relevantly, one of our recent studies using the SID task has found that the mild depressive participants showed a lower hit rate and a smaller CNV compared to the controls [53]. Taking a step further, this study examines whether these indexes would be sensitive to the depression level when individuals are interacting with a robot. Seeing that limited ERP studies have directly compared human–human and human–robot interactions, we did not formulate a priori hypotheses.

## Materials and methods

### Participants

Before the experiment, we conducted a priori power analysis using G*Power 3.1.7 [78, 79]. According to the effect size (${\eta}_{p}^{2}$ ≥ 0.051 for interaction effects) reported in one of our recent studies using a similar experimental design [53], the statistical power would be higher than 95% when there are 35 participants per group. A total of 70 adult participants were recruited from a large sample pool (approximately 500) in Shenzhen University. Participants completed the Beck Depression Inventory Second Edition (BDI-II) [80] during the recruitment stage. Based on BDI-II scores, two groups of individuals were invited, namely, a mild depressive symptom (MDS) group and a control group. The MDS group consisted of participants who scored larger than 13 (indicating mild depression), while the control group scored <13. The key exclusion criterion was any lifetime Axis I disorders according to Structured Clinical Interview for DSM-IV-TR Axis I Disorders, Research Version, Non-Patient Edition (SCID-I/NP) [81]. Other exclusion criteria included: (1) seizure disorder; (2) a history of head injury with possible neurological sequelae; (3) self-reported prior use of any psychoactive drugs; and (4) current alcohol or drug dependence. Among the students who met the above criteria, 70 individuals (35 with mild depression and 35 controls) were invited to participate in the experiment. According to their self-reports, all participants were medical-free at the time of the experiment.

On the day of the experiment, each participant was required to complete several questionnaires as soon as they come to the lab: (1) BDI-II; (2) the Trait form of Spielberger’s State-Trait Anxiety Inventory (STAI-T) [82]; (3) the Revised Social Anhedonia Scale (RSAS) [83]; (4) the Liebowitz’s Social Anxiety Scale (LSAS) [84]; (5) Rosenberg Self-esteem Scale (RSS) [85]; and (6) the “hedonic attitudes” subscale of Robot Acceptance Scale (RAS) [86], which measures how much a participant likes robots. As shown in Table 1, no significant difference was found between these two groups with respect to gender, age, handedness, and hedonic attitudes to robots, while the MDS group scored higher than the controls in depression, trait anxiety, social anhedonia, and social anxiety, but lower in self-esteem. Written informed consent was obtained prior to the experiment. The study was approved by the Ethics Committee of Shenzhen University.

**Table 1:** Demographic characteristics of the participants (mean and standard deviation).

Items	Control group (*n* = 35)	Mild depressive symptom (MDS) group (*n* = 35)	Control vs. MDS
Gender (male/female)	17/18	17/18	
Age (years)	19.6 (1.1)	19.9 (1.7)	*t* = −0.8, *P* = 0.427
Handedness, right/left	35/0	35/0	
BDI-II	5.6 (3.9)	19.1 (5.9)	*t* = −11.0, *P* < 0.001***
STAI-T	39.7 (9.1)	53.2 (8.1)	*t* = −6.5, *P* < 0.001***
RSAS	10.6 (4.3)	14.9 (6.9)	*t* = −3.1, *P* = 0.003**
LSAS	47.2 (17.5)	60.3 (19.5)	*t* = −2.9, *P* = 0.005**
RSS	27.0 (3.9)	23.3 (4.5)	*t* = 3.6, *P* = 0.001**
RAS	48.3 (9.3)	45.4 (10.9)	*t* = 1.2, *P* = 0.237

*BDI-II* the Beck Depression Inventory Second Edition, *STAI-T* the Trait form of Spielberger’s State-Trait Anxiety Inventory, *RSAS* the Revised Social Anhedonia Scale, *LSAS* Liebowitz’s Social Anxiety Scale, *RSS* Rosenberg Self-esteem Scale, *RAS* the hedonic factor of Robot Acceptance Scale.

**P < 0.01, ***P < 0.001.

### Experimental design and stimuli

A life-sized humanoid robot Karl was used in this study (version 1088, Guangzhou, China). Karl could respond to simple oral messages with relevant body gestures and screen-based facial expressions. For example, if somebody tells Karl: “you are so cool,” it would reply: “your praise is making me shy,” move hands up, and show a facial expression of embarrassment. Screen-based robot faces have several advantages compared to traditional mechanical faces, such as a wider variety of facial emotions and less cost of construction [87].

This experiment used 36 human and 36 robot facial expression pictures, with each cue condition (reward/punishment/neutral) having 12 of each kind of picture. Human facial pictures were taken from two undergraduate students (one male and one female) from the first author DZ’s lab. Robot facial pictures were taken from the robot Karl. Thirty volunteers (15 males; aged 22.3 ± 1.9 years; BDI scored 4.0 ± 3.8) assessed the emotional valence and arousal of these pictures on 9-point scales (Table 2); none of them participated in the formal experiment. Repeated-measures analyses of variances (ANOVAs) were performed on the valence (from 1: very negative to 9: very positive) and arousal (from 1: very low to 9: very high) ratings, respectively, with feedback provider (human vs. robot) and cue valence (reward vs. punishment vs. neutral) as two within-subject factors.

**Table 2:** Valence and arousal of pictures on a 1-to-9-point scale (mean and standard deviation).

Item	Human			Robot		
	Reward	Neutral	Punishment	Reward	Neutral	Punishment
Valence	6.5 (0.3)	4.5 (0.2)	3.5 (0.3)	6.5 (0.4)	4.6 (0.2)	3.3 (0.4)
Arousal	5.7 (0.3)	4.1 (0.3)	4.0 (0.2)	5.7 (0.4)	4.2 (0.2)	4.1 (0.3)

The results show that the main effect of cue valence was significant on valence rating (*F*(2,22) = 561.4, *P* *<* 0.001, ${\eta}_{p}^{2}=0.981$; reward [6.5 ± 0.3] > neutral [4.6 ± 0.2] > punishment [3.4 ± 0.4], pairwise *P*s ≤ 0.001) and arousal rating (*F*(2,22) = 284.5, *P* *<* 0.001, ${\eta}_{p}^{2}$ = 0.963; reward [5.7 ± 0.3] > neutral [4.1 ± 0.2]/punishment [4.2 ± 0.3], pairwise *P*s ≤ 0.001). Neither the main effect of feedback provider (*F* ≤ 1.6, *P* ≥ 0.229) nor the interaction effect (*F* ≤ 2.2, *P* ≥ 0.135) was significant, indicating that human and robot facial pictures provide comparable emotional experiences.

### Experimental procedure

Prior to the task, participants were invited to interact with the robot Karl, as well as the two undergraduate students whose facial pictures were used as experimental materials, for 5 min. Then their individual threshold of visual RT was assessed using a simple RT task (averaged across 30 trials), which was used to determine the target presentation time in the formal task (see below). After that, participants practiced in the human feedback condition and the robot feedback condition for ten trials, respectively. The practice trials and the formal task shared the same temporal structure. The only difference was that we used video clips in practice trials to enhance the experience of social interaction; in contrast, stationary pictures were employed in the formal task. This was because the ERP technique has a time resolution of milliseconds (much shorter than the presentation of video clips); accordingly, stationary pictures rather than video clips are more suitable for ERP research [88].

**Fig. 1:**
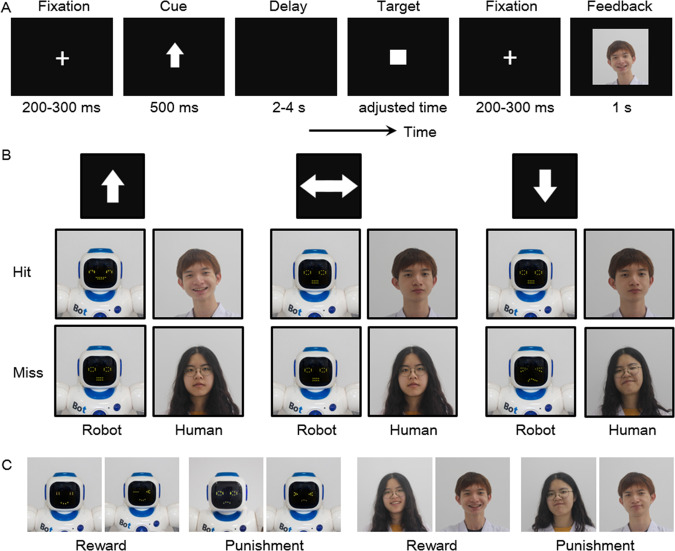
Experiment programs. **A** An exemplar trial in the reward condition. **B** The relationship between each kind of cue and feedback (for hits or misses). The two undergraduate students in the picture were from the first author DZ’s research group and have given their consent for these materials to appear in academic journals. **C** Examples of all kinds of positive and negative faces.

The formal SID task consisted of two human feedback and two robot feedback blocks. Each block consisted of 36 rewards, 36 neutral, and 36 punishment-cued trials, the order of which was pseudorandomly determined. The formal task (432 trials in total) lasted for ~40 min. The sequence of these two kinds of blocks was counterbalanced across participants. An overview of the trial structure is shown in Fig. 1. In both human feedback and robot feedback blocks, participants first saw a cue (i.e., the anticipation stage) indicating a potential reward (an upward arrow), punishment (a downward arrow), or neither (neutral condition: a horizontal arrow) for 500 ms. This cue was followed by a delay for a varied duration ranging from 2 to 4 s. The target stimulus then appeared, and participants were required to press the space button on a keyboard as quickly as possible to gain reward or avoid punishment. If participants’ RT was shorter than the duration of the target presentation, the ongoing trial would be labeled as a “hit” trial, otherwise, it would be labeled a “miss” trial. The target presentation time was initially set according to each participant’s behavioral performance in the simple RT task, then it was adjusted (±10 ms) in a trial-by-trial manner to keep the hit rate at ~50% [53, 89].

After the target presentation, each participant received outcome feedback for 1 s (i.e., the consumption stage). In the reward condition of human feedback blocks, successful hitting the target would lead to a picture of a smiling human face (indicating positive social evaluation), while missing the target would lead to a neutral human face; in the neutral condition, the feedback was always a neutral human face regardless of hit or miss; finally, in the punishment condition, successful hitting the target would lead to a picture of the neutral human face, while missing the target would lead to a human face with contempt (indicating negative social evaluation). These settings were consistent with the classic monetary incentive delay (MID) task, in which missing the target in the reward condition leads to no punishment, while hitting the target in the punishment condition leads to no reward [90, 91]. The same settings were used in robot feedback blocks, except that pictures of human faces were replaced by pictures of robot faces.

### Electroencephalographic (EEG) recording and analysis

Brain electrical activity was recorded by a 32-channel wireless amplifier with a sampling frequency of 250 Hz (NeuSen.W32, Neuracle, Changzhou, China). Data were online recorded referentially against the left mastoid and off-line re-referenced to average activities over the scalp. EEG data were collected with electrode impedances kept below 10 kΩ. Ocular artifacts were removed from EEGs using a regression procedure implemented in NeuroScan software (Scan 4.3: NeuroScan, Inc., Herndon, VA).

This study focused on the anticipation stage and the consumption stage of reward processing, corresponding to the ERPs evoked by cues and those evoked by feedback. The recorded EEG data were filtered (half-amplitude cutoff: 0.1–30 Hz) and segmented beginning 200 ms prior to stimulus (cue/feedback) onset. The cue-evoked ERP epochs lasted from −200 ms to 2.5 s while the feedback-evoked epochs lasted from −200 to 800 ms. All epochs were baseline-corrected with respect to the mean voltage over the 200 ms preceding stimulus onset, followed by averaging for each experimental condition. Epochs containing artifacts with peak-to-peak deflection exceeding ±100 μV were rejected. This procedure deleted 4.8 ± 2.2 trials per condition. The numbers of remaining trials for further analyses are reported in supplementary materials (Supplementary Table S1).

The electrode sites and time window for each ERP component were selected before data analysis based on prior knowledge. The main reference was one of our previous ERP studies which also used the SID task [53]. For cue-evoked ERPs, this study focused on the CNV elicited by reward, neutral, and punishment cues in human feedback and robot feedback blocks. The mean amplitude of the CNV was calculated using the arithmetic average of the electrode sites in the mid-frontocentral area (including Fz, FCz, FC1, and FC2) within a time window of 750–2500 ms post cue onset [92]. For feedback-evoked ERPs, we focused on the FRN and P3 elicited by feedback for hits or misses in human feedback and robot feedback blocks. The mean amplitude of the FRN was calculated using the arithmetic average, also at electrode sites in the mid-frontocentral area (i.e., Fz, FCz, FC1, and FC2), within a time window of 200 to 300 ms post feedback onset [93]. Finally, the mean amplitude of the P3 was calculated using the arithmetic average at electrode sites in the mid-parietal area (i.e., Pz, P3, and P4) within a time window of 300–600 ms post feedback onset [94, 95].

### Statistics

Repeated-measures ANOVAs were used to analyze the behavioral and ERP measures. Regarding the anticipation stage (i.e., cue presentation), a 2 × 3 × 2 mixed ANOVA was applied with feedback provider (human vs. robot) and cue valence (reward vs. punishment vs. neutral) as two within-subject factors and group (MDS vs. control) as the between-subject factor. The same model was applied to the consumption stage (i.e., feedback presentation), except that feedback valence (hit vs. miss) was added as the third within-subject factor (that is, a 2 × 3 × 2 × 2 mixed ANOVA). In addition, considering that anxiety and depressive symptoms are highly comorbid [96–98], we also performed ANOVAs on all the above dependent variables with trait anxiety as a covariate (see Supplementary Materials).

Descriptive data are presented as mean ± standard deviations, unless otherwise specified. The significance level is set at *P* = 0.05. Significant interactions were analyzed using a simple effects model. Partial eta-squared (${\eta}_{p}^{2}$) values are provided to demonstrate effect size where appropriate. For the sake of brevity, below we report significant interactions only when the group factor is involved. Other significant interactions could be found in supplementary materials.

## Results

### Behavioral results

#### Hit rate

**Fig. 2:**
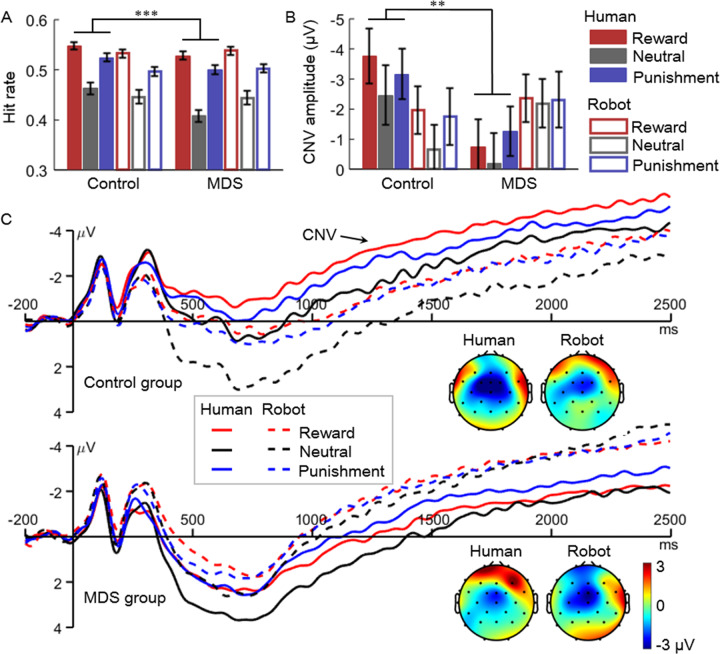
The results of hit rate and the contingent-negative variation (CNV). **A** Hit rate in the mild depressive symptom (MDS) and control groups. **B** Amplitudes of the cue-evoked CNV. Error bars indicate two standard errors. **P < 0.01, ***P < 0.001. **C** The CNV waveforms and topographic maps. The CNV waveforms were averaged across electrodes of Fz, FCz, FC1, and FC2. The CNV topographies were averaged across a time window of 750–2500 ms post cue presentation.

The main effect of the group was significant (*F*(1,68) = 25.3, *P* < 0.001, ${\eta}_{p}^{2}$ = 0.271; control vs. MDS = 50.2 ± 7.0% vs. 48.7 ± 7.7%). The main effect of cue valence was also significant (*F*(2,136) = 55.1, *P* < 0.001, ${\eta}_{p}^{2}$ = 0.448; reward (53.7 ± 4.7%) >punishment (50.6 ± 5.3%) >neutral (44.1 ± 8.0%), pairwise *P*s < 0.001). Moreover, the interaction of feedback provider × group was significant (*F*(1,68) = 38.2, *P* < 0.001, ${\eta}_{p}^{2}$ = 0.360; Fig. 2A); simple effect analysis reveals that the control group showed a higher hit rate (51.2 ± 6.9%) than the MDS group (47.9 ± 7.6%) in the human condition (*F*(1,68) = 57.3, *P* < 0.001, ${\eta}_{p}^{2}$ = 0.457), but not in the robot condition (*F* < 1; control vs. MDS = 49.2 ± 6.8% vs. 49.5 ± 7.7%).

#### RT

The RT was averaged across hit trials in each condition [53, 54]. The main effect of cue valence was significant (*F*(2,136) = 12.4, *P* < 0.001, ${\eta}_{p}^{2}$ = 0.154; rewards (216.4 ± 19.1 ms) < punishment (218.0 ± 20.5 ms) = neutral (219.3 ± 21.0 ms), pairwise *P*s ≤ 0.002).

### ERP data

#### Cue-evoked CNV

The main effect of cue valence was significant (*F*(2,136) = 8.6, *P* = 0.001, ${\eta}_{p}^{2}$ = 0.112; reward (−2.21 ± 3.54 μV) = punishment (−2.12 ± 3.50 μV) > neutral (−1.38 ± 3.78 μV), pairwise *P*s ≤ 0.007). Moreover, the two-way interaction of feedback provider × group was significant (*F*(1,68) = 37.6, *P* < 0.001, ${\eta}_{p}^{2}$ = 0.356; Fig. 2B, C); simple effect analysis reveals that the control group (−3.14 ± 4.77 μV) exhibited a larger (i.e., more negative-going) CNV than the MDS group (−0.74 ± 5.87 μV) in the human condition (*F*(1,68) = 8.8, *P* = 0.004, ${\eta}_{p}^{2}$ = 0.114), but not in the robot condition (*F*(1,68) = 1.4, *P* = 0.235, ${\eta}_{p}^{2}$ = 0.021; control vs. MDS = −1.46 ± 3.85 vs. −2.29 ± 5.96 μV).

Seeing that the hit rate and the CNV amplitude showed a similar pattern of results (i.e., a significant feedback provider × group interaction), we tested the two-tailed Pearson correlation between the above two indexes. This follow-up analysis showed that the hit rate and CNV amplitude were negatively correlated in both feedback conditions (*r* = −0.245 ~ −0.474, *P* = 0.041–0.0004, corrected for multiple comparisons using the false discovery rate method; Table 3). Again, please note that the CNV is a negative-going component; thus, these negative correlations indicate that the CNV amplitude increased as a function of the hit rate.

**Table 3:** Correlation between hit rate and CNV amplitude (*n* = 70).

Feedback provider	Reward			Neutral			Punishment		
	*r*	*p*	*p*_cor_^a^	*r*	*p*	*p*_cor_^a^	*r*	*p*	*p*_cor_^a^
Human	−0.452	<0.001	0.001	−0.273	0.022	0.044	−0.319	0.007	0.021
Robot	−0.474	<0.001	<0.001	−0.245	0.041	0.041	−0.404	0.001	0.004

^a^Corrected for multiple comparisons using the false discovery rate method.

#### Feedback-evoked FRN

The main effects of feedback provider (*F*(1,68) = 7.8, *P* = 0.007, ${\eta}_{p}^{2}$ = 0.103; human vs. robot = 0.36 ± 3.63 vs. 0.86 ± 3.46 μV), cue valence (*F*(2,136) = 14.3, *P* < 0.001, ${\eta}_{p}^{2}$ = 0.174; reward (0.94 ± 3.73 μV) = punishment (0.76 ± 3.35 μV) < neutral (0.14 ± 3.52 μV), pairwise *P*s ≤ 0.002), and feedback valence were significant (*F*(1,68) = 74.7, *P* < 0.001, ${\eta}_{p}^{2}$ = 0.524; hit vs. miss = 1.00 ± 3.57 vs. 0.22 ± 3.49 μV).

**Fig. 3:**
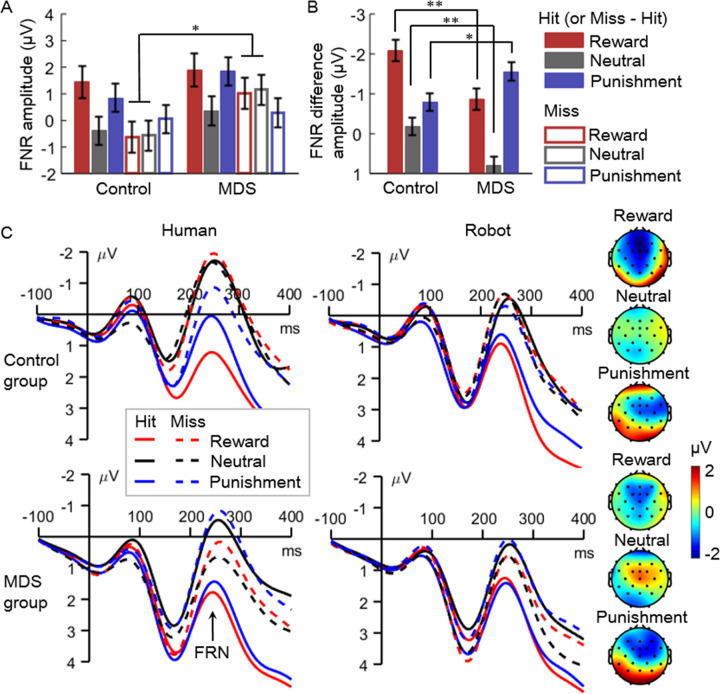
The results of feedback-related negativity (FRN). **A** Amplitudes of the FRN in different conditions. **B** Difference amplitudes of the FRN (miss subtracting hit trials). Error bars indicate two standard errors. *P < 0.05, **P < 0.01. **C** The FRN waveforms and topographies. The FRN waveforms were averaged across electrodes of Fz, FCz, FC1, and FC2. The FRN topographies were created according to difference amplitudes (miss subtracting hit trials) averaged across a time window of 200–300 post feedback.

The interaction of feedback valence × group was significant (*F*(1,68) = 6.8, *P* = 0.011, ${\eta}_{p}^{2}$ = 0.091). Further, the three-way interaction of cue valence × feedback valence × group was also significant (*F*(2,136) = 9.5, *P* < 0.001, ${\eta}_{p}^{2}$ = 0.123; Fig. 3B). To break down this three-way interaction, we examined the feedback valence × group interaction for reward, punishment, and neutral cues, respectively (Fig. 3A). Results reveal a similar pattern of the feedback valence × group interaction for reward (*F*(1,68) = 10.4, *P* = 0.002, ${\eta}_{p}^{2}$ = 0.133) and neutral cues (*F*(1,68) = 11.2, *P* = 0.001, ${\eta}_{p}^{2}$ = 0.142): the MDS group showed a smaller (i.e., less negative-going) FRN than the controls in response to miss feedback (reward: *F*(1,68) = 4.1, *P* = 0.046, ${\eta}_{p}^{2}$ = 0.057, control vs. MDS = −0.59 ± 3.33 vs. 1.08 ± 3.55 μV; neutral: *F*(1,68) = 4.8, *P* = 0.032, ${\eta}_{p}^{2}$ = 0.066, control vs. MDS = −0.54 ± 3.57 vs. 1.22 ± 3.11 μV), but not hit feedback (*F* < 1; reward: 1.45 ± 3.58 vs. 1.90 ± 3.59 μV; neutral: −0.36 ± 3.26 vs. 0.36 ± 3.25 μV). In contrast, the feedback valence × group interaction was not significant for punishment cues; specifically, the two groups showed comparable FRN amplitudes in response to hit (*F*(1,68) = 1.8, *P* = 0.184, ${\eta}_{p}^{2}$ = 0.026; control = 0.84 ± 3.38 μV, MDS = 1.85 ± 2.90 μV) and miss feedback (*F* < 1; control = 0.05 ± 3.23 μV, MDS = 0.29 ± 3.17 μV).

#### Feedback-evoked P3

The main effects of all the four factors were significant, including group (marginally, *F*(1,68) = 3.9, *P* = 0.052, ${\eta}_{p}^{2}$ = 0.055; control vs. MDS = 1.55 ± 3.66 vs. 0.19 ± 3.57 μV), feedback provider (*F*(1,68) = 6.9, *P* = 0.011, ${\eta}_{p}^{2}$ = 0.092; human vs. robot = 1.16 ± 3.69 vs. 0.58 ± 3.64 μV), cue valence (*F*(2,136) = 52.7, *P* < 0.001, ${\eta}_{p}^{2}$ = 0.437; reward (1.44 ± 3.62 μV) = punishment (1.50 ± 3.44 μV) > neutral (−0.33 ± 3.66 μV), pairwise *P*s < 0.001), and feedback valence (*F*(1,68) = 36.3, *P* < 0.001, ${\eta}_{p}^{2}$ = 0.348; hit vs. miss = 1.32 ± 3.60 vs. 0.43 ± 3.69 μV).

**Fig. 4:**
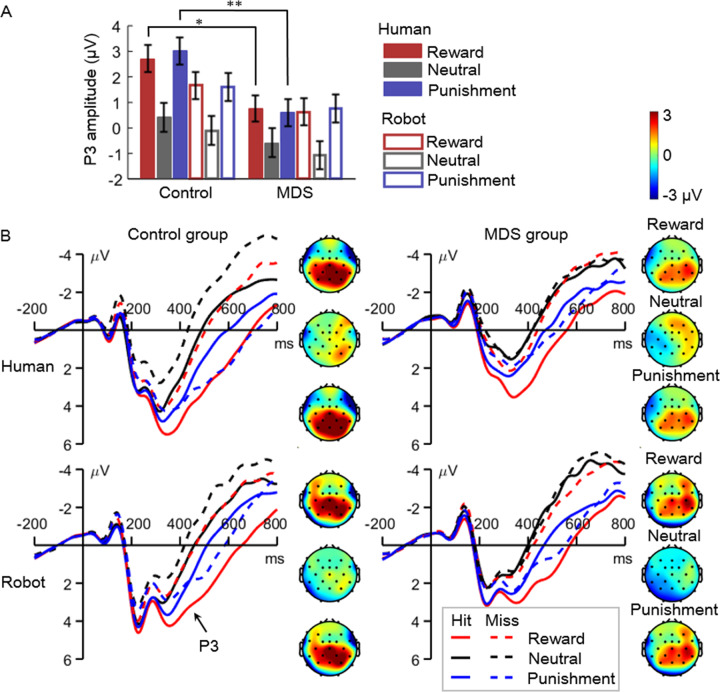
The results of feedback-evoked P3. **A** Amplitudes of the P3 in different conditions. *P < 0.05, **P < 0.01. **B** The P3 waveforms and topographies. The P3 waveforms were averaged across electrodes of Pz, P3, P4, CP1, and CP2. The P3 topographies were averaged across a time window of 300–600 ms post feedback.

The three-way interaction of feedback provider × cue valence × group was significant (*F*(2,136) = 5.8, *P* = 0.006, ${\eta}_{p}^{2}$ = 0.079; Fig. 4B). To break down this three-way interaction, we examined the feedback provider × group interaction for reward, punishment, and neutral cues, respectively (Fig. 4A). Results reveal a similar pattern of the feedback provider × group interaction for reward (*F*(1,68) = 4.0, *P* = 0.050, ${\eta}_{p}^{2}$ = 0.055) and punishment cues (*F*(1,68) = 8.8, *P* = 0.004, ${\eta}_{p}^{2}$ = 0.114): the MDS group showed a smaller P3 than the controls in the human feedback condition (reward: *F*(1,68) = 6.7, *P* = 0.012, ${\eta}_{p}^{2}$ = 0.090, control vs. MDS = 2.75 ± 2.91 vs. 0.84 ± 3.25 μV; punishment: *F*(1,68) = 9.1, *P* = 0.004, ${\eta}_{p}^{2}$ = 0.118, control vs. MDS = 2.91 ± 3.03 vs. 0.60 ± 3.36 μV), but not in the robot feedback condition (*F* ≤ 1.7, *P* ≥ 0.195; reward: 1.74 ± 3.34 vs. 0.72 ± 3.16 μV; punishment: 1.60 ± 3.43 vs. 0.75 ± 2.94 μV). In contrast, the feedback provider × group interaction was not significant in the neutral cue condition (*F* < 1); specifically, the two groups showed comparable P3 amplitudes in response to human (*F*(1,68) = 1.5, *P* = 0.230, ${\eta}_{p}^{2}$ = 0.021; control = 0.32 ± 3.42 μV, MDS = −0.65 ± 3.33 μV) and robot feedback (*F*(1,68) = 1.3, *P* = 0.263, ${\eta}_{p}^{2}$ = 0.018; control = −0.16 ± 3.39 μV, MDS = −1.06 ± 3.22 μV).

## Discussion

Seeing that machines and automations are becoming increasingly important in our society, understanding how people interact with these intelligent agents is necessary [99]. This study investigates whether social reward processing in depression would show different patterns for human–human and human–robot interactions in nonclinical populations. At the anticipation stage of social reward processing, the MDS group had a lower hit rate and a smaller cue-evoked CNV compared to the control group in the human condition but not the robot condition; then at the consumption stage, the MDS group had a smaller feedback-evoked P3 than the control group in the human condition but not the robot condition, after receiving reward and punishment (but not neutral) cues. Overall, these findings indicate that depressive symptoms are more likely to be associated with abnormalities in the anticipation and consumption stages of social reward processing during human–human interaction, compared to human–robot interaction.

### Anticipation stage

Since this experiment used a block design for two conditions (human vs. robot), participants were able to predict whether the upcoming feedback would come from a person or a robot in each block. As revealed by our results, this prediction has significantly modulated both task performance and neural signals among mild depressive participants. Specifically, when the evaluative feedback was expected to be delivered by a human companion, the depressive participants were less likely to hit the target stimulus within a given time window compared to the controls. Also, the CNV elicited by cue presentation (reflecting the preparation process for the forthcoming target) [64] was smaller among the depressive participants than the controls. These results are in line with our recently published data [53].

According to the literature, depression symptoms are closely associated with low anticipation of social rewards, which is one of the major characteristics of social anhedonia [7, 100]. In our opinion, the depressive participants had difficulties in conceiving and expecting social reward during their interactions with other people; consequently, they mobilized less effort to finish the follow-up task and thus provided worse performance [101–103]. Our interpretation is supported by the correlation between the hit rate and the CNV amplitude, which has also been observed in our previous research [53]. In contrast, when the feedback was expected to be delivered by the robot Karl, no between-group difference was detected in the hit rate or CNV amplitude. Therefore, we suggest that participants’ motivation to achieve social reward in this condition was not significantly affected by depression.

### Consumption stage

The effect of depression was significant at both the early, coarse phase (indexed by the FRN) and the late, deliberate phase (indexed by the P3) of social reward consumption [52, 54]. First, the depressive participants showed a smaller FRN elicited by miss feedback than the controls in the reward and neutral cue conditions. In both conditions, miss feedback indicates the omission of social reward. Thus, we suggest that the FRN finding indicates blunted sensitivity to social reward in depression [52, 102, 104]. In line with these results, previous studies have consistently reported that the FRN amplitude decreases as a function of depression severity in various reward-related paradigms [105, 106]. However, the relationship between depression and the FRN remained the same pattern regardless of whether participants were interacting with other persons or with the robot Karl. In our opinion, this was because the early phase of feedback evaluation is susceptible to the similarities in perceptual appearance between humans and human-like machines, that is, an effect of anthropomorphism (see “Introduction”).

More importantly, the effect of depression on the P3 component (indicating a more top-down phase of feedback evaluation) differentiated between human–human and human–robot interactions. That is, the P3 elicited by human feedback, but not robot feedback became smaller among the depressive participants than the controls in the reward and punishment cue conditions. While the P3 has been related to diverse cognitive operations [107, 108], many studies suggest that this component is closely associated with a motivational level in decision-making tasks, such that a larger P3 indicates the stronger motivational significance of the ongoing event [56, 71, 109–111]. For instance, a larger P3 was observed when participants were comparing their task performance with that of a disliked opponent’s, indicating stronger motivation to outperform that opponent [112]. Given this background knowledge, we propose a possible interpretation of the P3 finding: compared to the controls, mild depressive individuals’ motivation to provide better performance in a social scenario is less likely to be modulated by their expectation of receiving positive social feedback (or avoiding negative social feedback) from a human companion. In agreement with our explanation, Frey, Frank, & McCabe recently point out that depression negatively affects the ability in using social feedback to appropriately update future actions [101, 113]. In contrast, the motivational level reflected by P3 amplitude was unresponsive to depression when participants received feedback from the robot Karl.

As an alternative explanation, the P3 amplitude may indicate the amount of attentional resources during feedback processing. This explanation stems from the knowledge that the P3 component has been frequently related to attentional function [108, 114]. From this perspective, mild depressive participants might have allocated fewer attentional resources than the controls on human feedback in the reward and punishment cue conditions, possibly because they were pessimistic about receiving favorable feedback from humans. Future studies are awaited to determine between the “attentional” hypothesis and the “motivational” hypothesis, though both of them are based on the relationship between depression and feedback expectation.

## Conclusions and concluding remarks

In the science fiction *I, Robot* by Isaac Asimov, its protagonists argue that robots are actually more reliable and trustworthy than humans—many people in real life share the same belief [115, 116]. For instance, de Visser et al. reported that anthropomorphic robot agents were associated with greater trust resilience (i.e., a higher resistance to breakdowns in trust) than human agents [28, 117]. Relevantly, the current study discovers that social reward processing during human–robot interaction is less prone to be affected by depression compared to that during human–human interaction. That is, depressive participants held a more pessimistic expectation about getting social rewards and were less likely to be motivated by those rewards in the human condition, therefore they showed poorer task performance than the controls; nevertheless, the same was not true in the robot condition.

According to the clinical literature, depressive episodes are strongly associated with negative social experience (e.g., peer rejection in schools) in which people fail to receive social rewards [118–120]. Among depressive individuals, this kind of experience generates social evaluative concerns and accordingly inhibits the willingness to engage in social interactions [121–124]. As a result, depressive individuals perceive themselves to be less socially competent than ordinary people and underestimate the likelihood of obtaining favorable feedback (e.g., social approval) from other persons [7]. On the contrary, we suggest that interacting with robots may not be interfered with by these concerns, because people tend to trust the objectivity of robot feedback. From a theoretical perspective, our findings indicate that human–robot interaction not only has social rewarding properties, but is also safe from some risk factors associated with human–human interaction (e.g., social evaluative threats and peer pressure); depression symptoms interplay with these risk factors during human–human interaction, but not human–robot interaction. These findings may help understand to what extent classical theories of social psychology (which are based on human–human interaction) could be used to explain human–robot interaction [14, 22]. From a practical perspective, our findings address why companion robots significantly improve quality of life, mood, and loneliness for adults with depression, and highlights the value of this kind of robots as a useful tool in clinical practice [19, 46].

Below we list some limitations and future directions for follow-up research to consider. Most importantly, our task did not allow life-like communication between participants and different agents due to technical restrictions; instead, we provided human and robot facial expression pictures to participants as social feedback. Although face processing is a fundamental part of social interaction [125], the real-time exchange of verbal and gestural signals should have enhanced the ecological validity of this study. Thus, we encourage follow-up studies to re-examine the reliability of our findings in more ecological contexts, and to investigate the potential influence of social intelligence skills of robots (not just their appearance). Second, seeing that the robot Karl has an anthropomorphic appearance, future studies could try using robots with alternative designs (e.g., zoomorphic or caricatured) for practical reasons. Moreover, it should be noted that the current findings are derived from the SID task; using alternative paradigms associated with social reward processing (e.g., the Ultimatum Game or the Trust Game [126]) would help examine the generality of these findings. Finally, it would be interesting to explore the processing of robot feedback in other clinical conditions. For instance, Raffard et al. point out that patients with schizophrenia may have difficulties in recognizing and interpreting complex social cues from humans and human-like robots [47, 127]. Accordingly, we hypothesize that schizophrenia would affect social reward sensitivity in not only human–human but also human–robot interactions.

## Supplementary information

Supplementary materials

## Data Availability

All the data and code used in this study could be available by contacting the first author, Prof. Dandan Zhang (e-mail: zhangdd05@gmail.com).
