# Supplementary material for: I, robot: depression plays different roles in human–human and human–robot interactions
Source: Transl Psychiatry. 2021 Aug 21;11:438. doi: 10.1038/s41398-021-01567-5 (PMC8380250; doi:10.1038/s41398-021-01567-5)
Supplement: Supplementary file 1 — Supplementary materials [file 41398_2021_1567_MOESM1_ESM.docx]

Zhang et al.

## Supplementary Materials

I, robot: Depression plays different roles in human-human and human-robot interactions

**The number of remaining trials after artifact rejection**

There were 36 trials in each condition (432 trials in total). During data preprocessing, we excluded the epochs containing artifacts with peak-to-peak deflection exceeding ±100 μV. This procedure deleted 4.8 ± 2.2 trials per condition. The numbers of remaining trials for further analyses are listed in Table S1.

**Table S1. Valid trial numbers in each condition** (mean & standard deviation).

| Group | Human | | |  | Robot | | |
| --- | --- | --- | --- | --- | --- | --- | --- |
|  | reward | neutral | punishment |  | reward | neutral | punishment |
| Control | 32.0 (2.3) | 32.3 (2.2) | 31.1 (2.1) |  | 30.2 (1.9) | 31.3 (2.1) | 31.4 (2.2) |
| MDS | 30.8 (1.9) | 31.6 (2.0) | 30.4 (2.2) |  | 30.8 (2.1) | 30.7 (2.2) | 31.7 (2.3) |

MDS, mild depressive symptom

**Other significant effects in the event-related potential data**

*Feedback-evoked FRN*

The interaction effects of *feedback sender* × *cue valence* (*F*(2,136) = 5.1, *p* = 0.008, $\eta_{p}^{2}$= 0.069) and *cue valence* × *feedback valence* were significant (*F*(2,136) = 29.8, *p* < 0.001, $\eta_{p}^{2}$= 0.305). Also, the three-way interaction of *feedback sender* × *cue valence* × *feedback valence* was significant (*F*(2,136) = 7.2, *p* = 0.002, $\eta_{p}^{2}$= 0.096). To break-down the three-way interaction, we examined the *feedback sender* × *cue valence* in hit and miss trials, respectively. For miss trials, only the main effect of *feedback sender* was significant (*F*(1,69) = 13.1, *p* = 0.001, $\eta_{p}^{2}$= 0.160; human vs. robot = -0.11 ± 3.59 vs. 0.55 ± 3.38 μV). For hit trials, the main effect of *cue valence* [*F*(2,138) = 36.9, *p* < 0.001, $\eta_{p}^{2}$= 0.349; positive (1.68 ± 3.72 μV) and negative (1.35 ± 3.32 μV) < neutral (-0.01 ± 3.47 μV), pairwise ps < 0.001] as well as the two-way interaction was significant (*F*(2,138) = 10.0, *p* < 0.001, $\eta_{p}^{2}$= 0.127). Simple effect analysis reveals that the effect of *cue valence* was more significant for human feedback [*F*(2,68) = 42.0, *p* < 0.001, $\eta_{p}^{2}$= 0.553; positive (1.78 ± 3.88 μV) and negative (1.21 ± 3.39 μV) < neutral (-0.50 ± 3.24 μV), pairwise *p*s < 0.001] than robot feedback [*F*(2,68) = 9.6, *p* < 0.001, $\eta_{p}^{2}$= 0.221; positive (1.57 ± 3.57 μV) and negative (1.48 ± 3.28 μV) < neutral (0.50 ± 3.64 μV), pairwise *p*s ≤ 0.002].

*Feedback-evoked P3*

The interaction of *cue valence* × *feedback valence* was significant (*F*(2,136) = 36.6, *p* < 0.001, $\eta_{p}^{2}$= 0.350). While the P3 was larger for hit compared to miss trials in positive (*F*(1,68) = 86.8, *p* < 0.001, $\eta_{p}^{2}$= 0.561; hit vs. miss = 2.62 ± 3.43 vs. 0.27 ± 3.44 μV) and neutral cue conditions (*F*(1,68) = 6.9, *p* = 0.010, $\eta_{p}^{2}$= 0.093; hit vs. miss = 0.01 ± 3.60 vs. -0.68 ± 3.71 μV), its amplitude was larger for miss (1.69 ± 3.57 μV) compared to hit trials (1.31 ± 3.31 μV) in negative cue condition (*F*(1,68) = 4.0, *p* = 0.050, $\eta_{p}^{2}$= 0.055).

**ANOVAs with trait anxiety as a covariate**

In this part, we test whether the effect of depressive symptoms was influenced by the effect of trait anxiety.

## Behavioral results

*Hit rate*

When considering the trait anxiety as a covariate, the main effect of *group* was still significant (*F*(1,67) = 16.6, *p* < 0.001, $\eta_{p}^{2}$= 0.199). Moreover, the interaction of *feedback provider* × *group* was also significant (*F*(1,67) = 31.5, *p* < 0.001, $\eta_{p}^{2}$= 0.320).

*Reaction time*

No effect of mild depressive symptoms was found in the main test.

## ERP data

*Cue-evoked CNV*

When considering the trait anxiety as a covariate, the two-way interaction of *feedback provider* × *group* was still significant (*F*(1,67) = 43.7, *p* < 0.001, $\eta_{p}^{2}$= 0.395).

*Feedback-evoked FRN*

The interaction of *feedback valence* × *group* was still significant (*F*(1,67) = 5.6, *p* = 0.021, $\eta_{p}^{2}$= 0.077). Further, the three-way interaction of *cue valence* × *feedback valence* × *group* was also significant (*F*(2,134) = 9.1, *p* < 0.001, $\eta_{p}^{2}$= 0.120).

*Feedback-evoked P3*

The main effects of *group* was still significant (*F*(1,67) = 4.5, *p* = 0.038, $\eta_{p}^{2}$= 0.063). The three-way interaction of *feedback provider* × *cue valence* × *group* was also significant (*F*(2,134) = 7.8, *p* = 0.001, $\eta_{p}^{2}$= 0.105).
